# Response of soil fungal-community structure and function to land conversion to agriculture in desert grassland

**DOI:** 10.3389/fmicb.2024.1413973

**Published:** 2024-09-09

**Authors:** Peng Kang, Jinpeng Hu, Yaqing Pan, Xuan Qu, Yichao Ran, Chenxi Yang, Bingru Liu

**Affiliations:** ^1^School of Biological Science and Engineering, North Minzu University, Yinchuan, China; ^2^College of Pastoral Agriculture Science and Technology, Lanzhou University, Lanzhou, China; ^3^Shapotou Desert Research and Experiment Station, Northwest Institute of Eco-Environment and Resources, Chinese Academy of Sciences, Lanzhou, China

**Keywords:** desert grassland, land conversion, fungal community, network, functional groups

## Abstract

Land conversion to agriculture is an important factor affecting soil ecological processes in the desert grasslands of northern China. However, soil fungal-community structure and function in response to Land conversion remain unclear. In this study, desert grassland, artificial shrubland, and land conversion were investigated in the western part of the Mu Us Sandland (Yanchi, Ningxia; Dingbian, Shaanxi). We found that land conversion significantly increased soil total carbon, nitrogen, and phosphorus, and available phosphorous and potassium contents. In the early stage of conversion to agricultural (April), soil fungal operational taxonomic units and abundance-based coverage estimator were lower than those of dessert grasslands and shrubland plots and had significant correlations with pH, electric conductivity, and available phosphorus and potassium. The dominant phyla strongly correlated with soil physicochemical properties. Concomitantly, the relative abundance of Glomeromycota was significantly lower, and the complexity of the network in the land conversion plots was lower than that in the shrubland plots. In the late stage of land conversion (September), soil fungal operational taxonomic units and abundance-based coverage estimator were lower in the conversion plots than in the desert grassland plots, with more complex network relationships compared to the desert grassland or shrubland plots. Symbiotrophic groups, a functional group of desert grassland soil fungi, can be used as a predictor of environmental change; in addition, land conversion decreases the relative abundance of arbuscular mycorrhizal functional groups. Our study highlights the response of soil fungal communities and functions to human disturbances in desert grasslands. Considering the potential of land conversion to agriculture to influence soil secondary salinization, there is a need for continued observation of soil ecological health over the time continuum of land conversion to agriculture.

## 1 Introduction

Global climate change profoundly affects the structure and function of desert grassland (DG) ecosystems, where limited water availability has become a major barrier to restoration and reconstruction (Yang et al., 2021; Sun et al., 2022). In addition, human disturbances have increased the vulnerability of desert grassland ecosystems by, for example, the reduction of vegetation cover associated with land conversion to agriculture (spring clearing and fall harvesting) (Xu et al., 2020), or through further salinization of the soil by groundwater extraction for irrigation (Hu et al., 2021). The additional water and nutrient inputs to the soils of arid areas associated with land conversion to agriculture not only alter the soil’s physical properties but break the elemental balance of the soils in this region as well, thereby accelerating the irreversible impacts of the loss of soil nutrients such as soil organic matter and nitrogen (Li et al., 2020; Wang et al., 2023). Hence, there is a need for research on farmland conversion in the desert grasslands of northwestern China, as such farmland conversion is widespread in this region.

Soil fungal communities play an important role in regulating the operation of terrestrial ecosystems and researchers have extensively reported on the effects of land-use changes, such as afforestation and land conversion (Qiang et al., 2020; Luo S. et al., 2023; Luo X. et al., 2023). Thus, while afforestation increases fungal diversity, land conversion alters soil fungal community structure. Specifically, the richness of the fungal community increases with increasing time of conversion, and the diversity of the fungal community changes (Qiang et al., 2020). Further, it is worth noting that soil fungal communities are sensitive to changing climatic factors, especially precipitation and temperature. Seasonal variation-driven environmental changes in these variables profoundly affect soil fungal-community structure and function (Wang et al., 2020). For example, the functional groups of saprophytic fungi explain the effects of land use on soil physical and chemical properties in desert grasslands (Kang et al., 2023). The relationship between soil fungi and plant communities, and the drivers of soil fungal communities that differ between deserts and grasslands constitute another example (Wang et al., 2018). However, the effects of land conversion to agriculture on the community structure and function of soil fungi have been largely ignored.

While studying desert grasslands in northern China, to date researchers have not only focused on the interrelationship between soil fungal communities and plant communities (Wang et al., 2018), additionally, they have clarified the response of soil fungal communities to environmental changes in these ecosystems (Cui et al., 2018). In an increasing number of studies, the strength of network relationships has been used to prove the response of fungal taxa to climate and soil environment changes. However, most studies focus on the changes of nodes and edges in the network, because the degree of nodes often reflects the importance of microbial taxa in the network relationship, just like a transportation hub. Positive links and negative edges can also intuitively represent the relationship between nodes. In recent studies, the calculation and extraction of subnetwork data not only deepened our understanding of network complexity, but also clarified the important role that the network complexity index (NCI) plays in explaining environmental change, which represents the complex interactions between microbes, including symbiosis, competition, predation, and other relationships (Luo S. et al., 2023; Luo X. et al., 2023). Therefore, it is reasonable to assume that elucidating the response characteristics of fungal network NCI to soil environmental changes in the context of land conversion in desert grasslands is also of positive significance.

In addition, the fungal functional guild is not only a way to analyze the lifestyle of identified taxa (Nguyen et al., 2016), but also serves as a class of groups for evaluating the response of fungal taxa to soil environmental changes (Kang et al., 2023; Liu et al., 2023). Further, the nutrient acquisition strategies and ecological functions of the fungal taxa can be represented in functional guilds (Nguyen et al., 2016). Especially in the west of the Mu Us Sandland in northern China (Yanchi, Ningxia; Dingbian, Shaanxi), the dynamic response of the soil fungal-community structure and functional changes to land conversion in desert grassland ecosystems, remain unclear. Therefore, this study investigated desert grassland, shrubland, and converted land soils, and analyzed soil physicochemical properties and fungal ITS high-throughput sequencing in April and September, respectively, to address the following scientific questions: (1) which soil environmental factors have a positive response to soil fungal community structures under land conversion; (2) what are the changes of soil fungal functional guilds under conversion to agriculture?

## 2 Materials and methods

### 2.1 Study area

The study area is located in the west of Ordos and the Mu Us sandy land in the east, which belongs to the desert grassland ecosystem. The predominant plants in the study area are *Sophora alopecuroides*, *Peganum harmala*, *Caragana korshinkii*, *Tamarix chinensis*, and *Nitraria tangutorum*, most of which are xerophytes. The average annual temperature in the past 5 years was 7.9°C and the average annual precipitation was 255.35 mm. Maize cultivation has become a major approach to improve the economic income of local farmers within the region. However, arable land is limited and conversion of desert grassland for maize cultivation is widespread. The desert grassland is converted and then fertilized to increase nitrogen, phosphorus, and potassium inputs (CH_4_N_2_O: 450 kg/ha year^−1^; N-P_2_O_5_-K_2_O: 300 kg/ha year^−1^); this is followed by another application of fertilizers at the same concentration as the first time in June and August, and approximately three times throughout the year. The corn field is watered mainly by pumping groundwater approximately 4–5 times according to the actual precipitation of the year.

### 2.2 Soil sample collection

**Figure 1:**
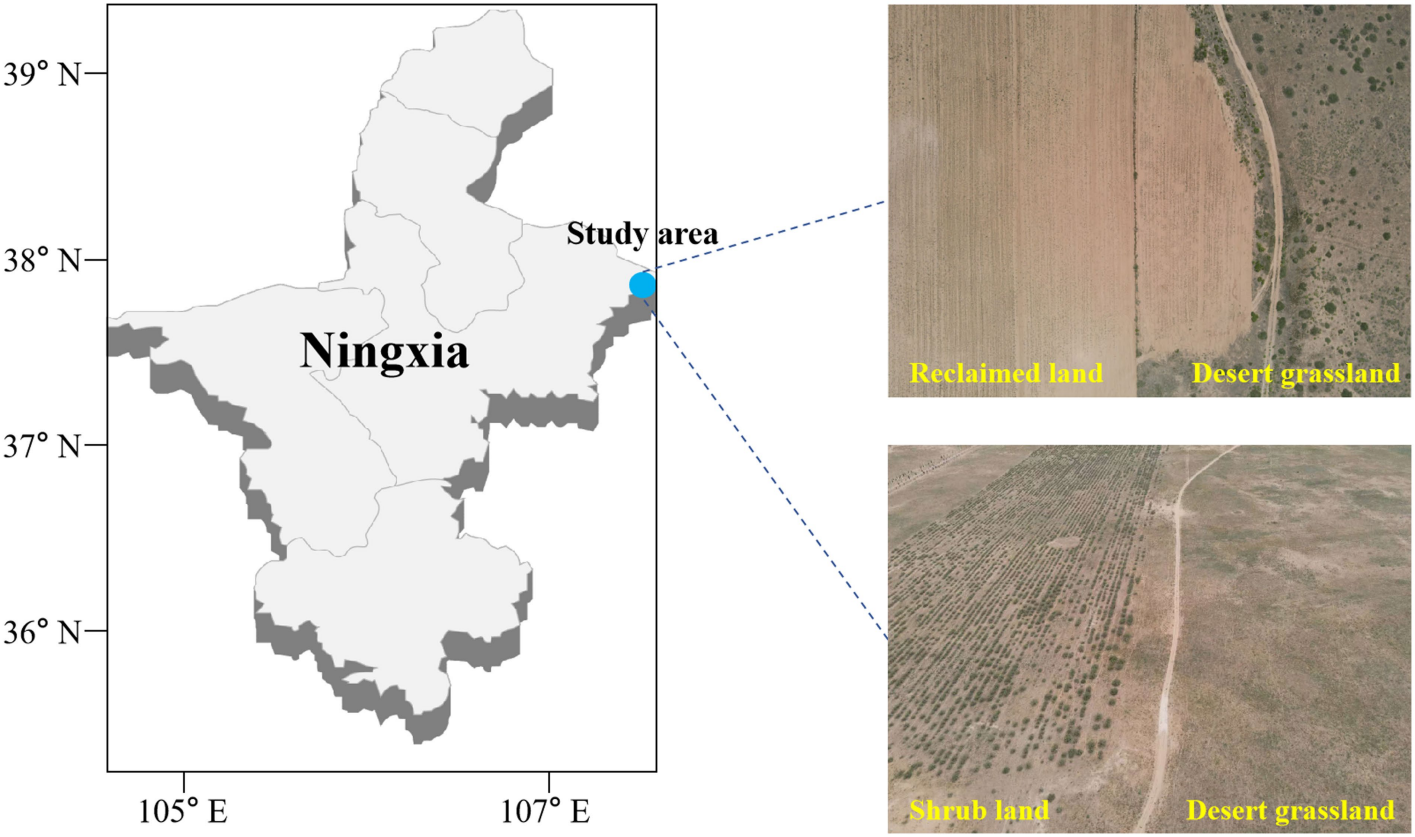
Sampling sites in the western part of the Mu Us Sandland at the junction of Yanchi County, Ningxia and Dingbian County, Shaanxi.

This study was conducted on desert grassland (DG, *S. alopecuroides* community) and artificial shrub land (SL, *C. korshinkii* community) that had been maintained for over 8 years, and land conversion to agricultural land (RL, a former *S. alopecuroides* community converted to agriculture in March 2021 and planted with maize). Among the studied areas, the DG and SL each cover an area exceeding 10 km^2^, while the RL is approximately 5 km^2^. Within each study area, comprising DG, SL, and RL, nine sampling sites were selected, with the distance between each sampling point exceeding 200 meters (Figure 1). To clarify the effect on soil physicochemical properties under seasonal variations, we obtained samples in April (average temperature (MMT), 10.1°C and average precipitation (MMP), 29.3 mm) and September (average temperature, 17.9°C and average precipitation, 55.5 mm) 2021. In each sampling site, 100 m^2^ was randomly divided and 0–10 cm of soil was collected by the 5-point sampling method as a representative of that sampling site (Pan et al., 2021). After surface impurities were removed, two soil samples were collected at each sample point and transferred to the lab using an ice box. One of the soil samples was used for physical and chemical characterization after being sieved through a 2-mm sieve. The other was used for ITS high-throughput sequencing of fungi. The latter process was carried out in a sterile environment. PBS buffer was added to the soil sample and shaken to remove roots and other impurities. Then, the soil sample was centrifuged at 6000 *g* for ten minutes at 4°C, and the supernatant was removed to obtain the desired soil sample.

### 2.3 Analysis of soil physical and chemical properties

The soil water content (SWC) was determined using the weighing method, and soil pH and electrical conductivity (EC) were determined at a soil-water ratio of 1:2.5 (Bao, 2000). The sieved soil was introduced into a carbon and nitrogen analyzer for the determination of total carbon (TC) and nitrogen (TN) (CN 802 Carbon Nitrogen Elemental Analyzer, Shanghai, China). The content of total phosphorus (TP) in the soil was determined using the sulfate-molybdenum dysprosium resistance colorimetric method. Soil total organic carbon (TOC) content was determined through potassium dichromate oxidation spectrophotometry (Jiang et al., 2024). The content of soil alkali-hydrolysable nitrogen (AN) was determined using the zinc-ferrous sulfate reduction method (Wu et al., 2021). The soil available phosphorus (AP) was determined using the sodium hydrogen carbonate-molybdenum-dysprosium anti spectrophotometric method, and the soil available potassium (AK) was determined using ammonium acetate flame spectrophotometry (Bao, 2000).

### 2.4 High-throughput sequencing of soil fungi and data analysis

DNA was extracted from screened soils using a 16-alkyltrimethylammonium bromide kit, and the ITS1-2 gene region of 54 eligible samples was amplified with primers ITS1 (5’-CTTG GTCA TTTA GAGG AAGT AA-3′) and ITS2 (5’-GCTG CGTT CTTC ATCG ATGC-3′) (Cheng et al., 2022). After PCR amplification, Illumina NovaSeq PE250 platform was used for sequencing. An average of 86,200 tags were measured per sample, and an average of 84,713 valid data were obtained for quality control. Subsequently, we standardized the data from each sample by subsampling based on the sample which has a minimum sequence depth. All subsequent analyses are based on this program. Clustering sequences into operational taxonomic units (OTUs) with 97% identity yields 5,893 OTUs. Fungal raw data for 54 soil samples from this study were submitted to the NCBI database (PRJNA1064665).

**Figure 2:**
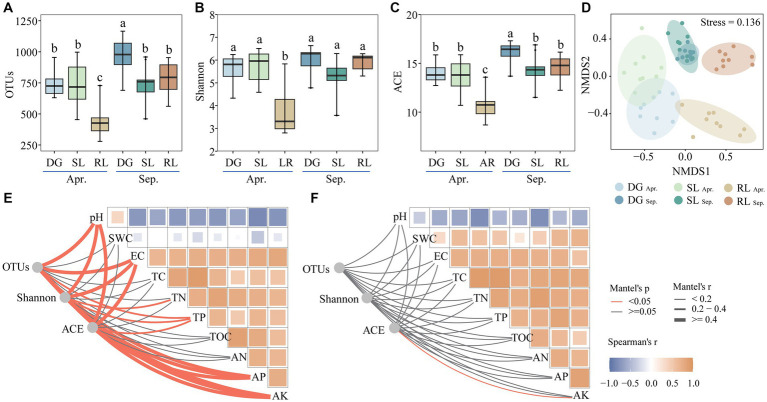
Alpha diversity **(A–C)**, NMDS **(D)** and Mantel’s test **(E)** in April, **(F)** in September under seasonal variation and land conversion in desert grassland. DG, desert grasslands plots; SL, shrub land plots; RL, land conversion plots. Apr, April; Sep, September.

**Figure 3:**
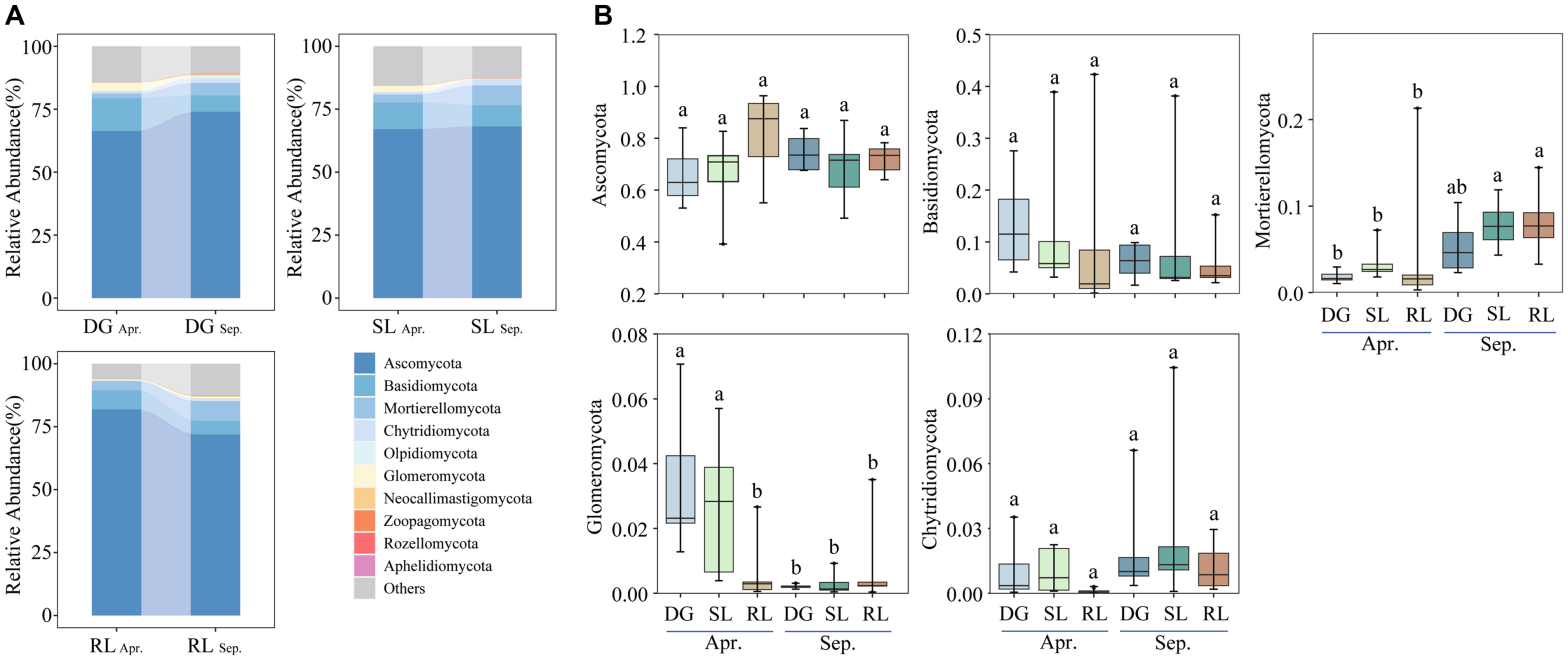
Relative abundance of dominant fungal phyla **(A)** top10 phyla; **(B)** top5 phyla under seasonal variation and land conversion in desert grassland. DG: desert grasslands plots; SL: shrub land plots; RL: land conversion plots. Apr: April; Sep: September.

Soil physicochemical characteristics of the three sampling sites in April and September were statistically analyzed using ANOVA (Supplementary Figure S1). The OTUs, and Shannon and ACE indices for the fungal communities were calculated using QIIME (V1.9.1) software (Caporaso et al., 2012). Bray-Curtis was used to calculate the beta diversity index of fungal communities (Kang et al., 2022). The Mantel test, implemented via the linkET package in R software (v 4.1), was employed to assess the Spearman correlation between the matrix of fungal alpha diversity (e.g., OTUs, and Shannon and ACE indices) and soil physicochemical properties. During this process, the default mode was selected for other parameters (Sunagawa et al., 2015) (Figure 2). The dominant fungal phyla (average relative abundance >1%) between the groups were presented by the “circlize” package (Pan et al., 2021). In addition, the differences in the relative abundance of dominant phyla were also analyzed by ANOVA and shown using the “ggplot2” software package (Figure 3). To clarify the response of soil fungal phyla to soil environmental changes in the three sampling plots, we determined the relationship between dominant phyla and environmental factors by Spearman’s analysis (Pan et al., 2022). After the fungal community was standardized by the Hellinger method, a non-metric multidimensional scaling (NMDS) analysis based on Bray-Curtis distance was performed. Meanwhile, the redundancy analysis (RDA) analysis further demonstrated the relationship between dominant fungal phyla and environmental factors at the OTU level in desert grassland (Supplementary Figure S2).

**Figure 4:**
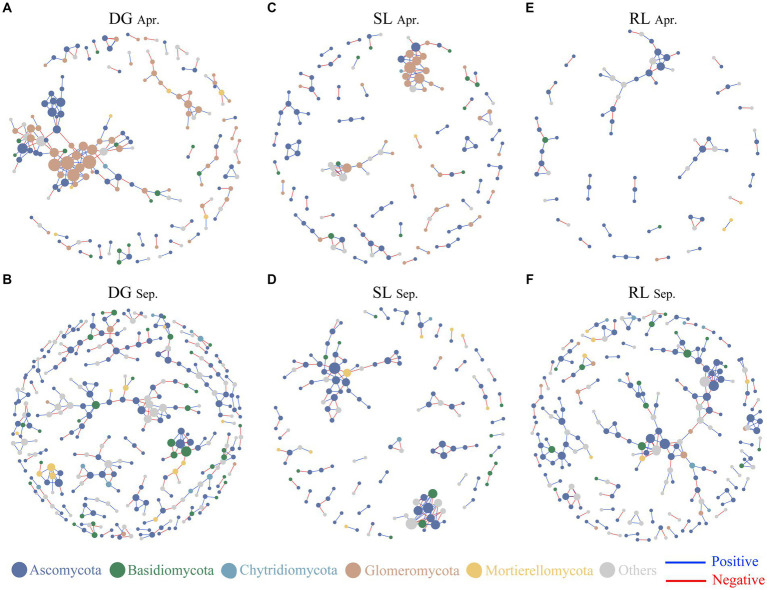
Soil fungal networks under seasonal variation and land conversion in desert grassland. The color of the nodes indicates different fungal phyla. DG, desert grasslands plots **(A, B)**; SL, shrub land plots **(C, D)**; RL, land conversion plots **(E, F)**. Apr, April; Sep, September.

**Figure 5:**
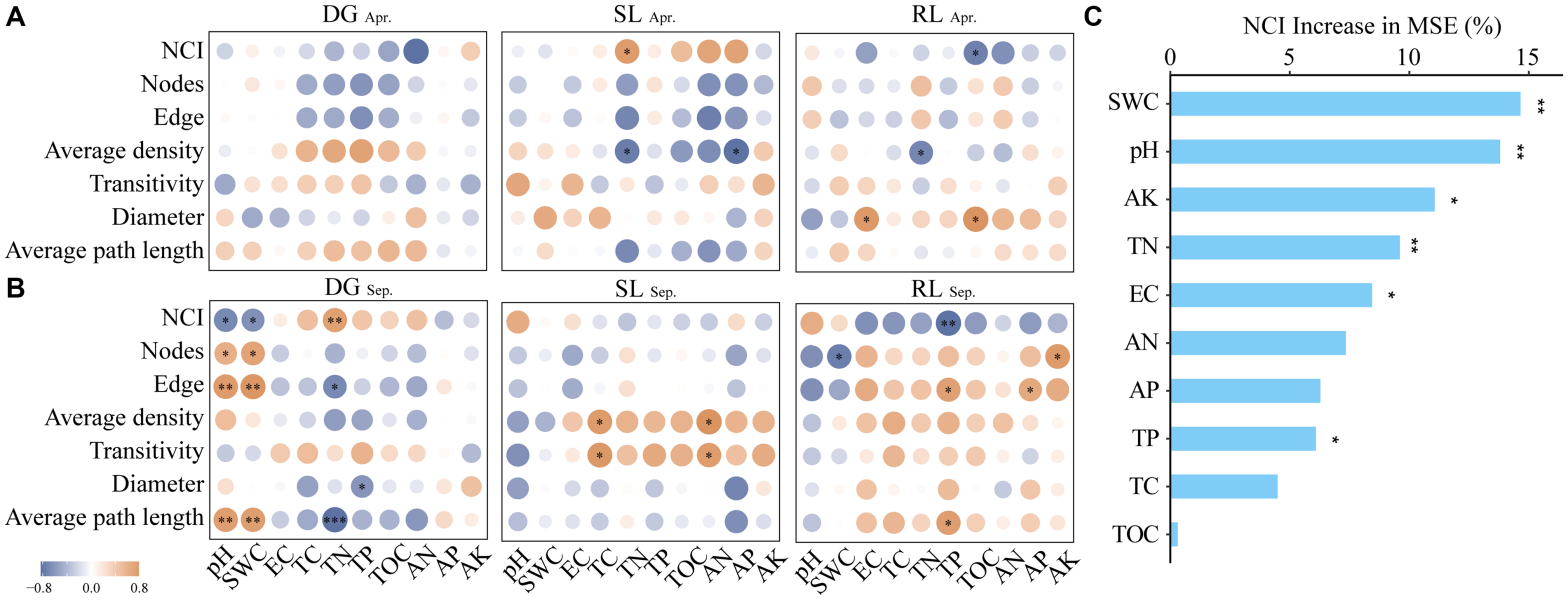
Correlation analysis between NCI and environmental factors **(A)** Apr; **(B)** Sep and random forest analysis **(C)** under seasonal variation and land conversion in desert grassland. DG, desert grasslands plots; SL, shrub land plots; RL, land conversion plots. Apr, April; Sep, September.

In this study, the correlation of the absolute abundance table of fungi at the OTU level in April and September in the three sampling sites was subjected to network analysis (with a correlation threshold of |r| > 0.9). In detail, using the Molecular Ecological Network Analysis Pipeline (MENAP, ieg4.rccc.ou.edu/mena/) based on Random Matrix Theory (RMT), we calculated the Spearman correlation for OTUs that had a detection rate exceeding 77% and underwent centered log-ratio transformation, provided they were present in at least 7 out of 9 replicates, and the *p*-value of the chi-square test for Poisson distribution was specified to be greater than 0.05. After obtaining the node and edge data, the information was visualized using Cytoscape software (v 3.7.1) (Figure 4). After that, sub-network topological features such as node, edge, average density, transitivity, diameter, and average path length were generated for each sample from the global microbial network using the subgraph() function in the “igraph” package (Ma et al., 2016; Manning et al., 2018). The PCA1 values were further obtained by principal component analysis of the sub-network data to characterize the complexity of the fungal network (NCI) (Zhang et al., 2018; Qiu et al., 2021); where diameter and average path length were calculated by inverse form (X-1). Spearman’s correlation analysis of NCI, subnetwork data, and soil physicochemical properties was also demonstrated for each sample plot. We further identified environmental factors with high explanatory rates for NCI by random forest analysis (Jiao et al., 2022) (Figure 5).

**Figure 6:**
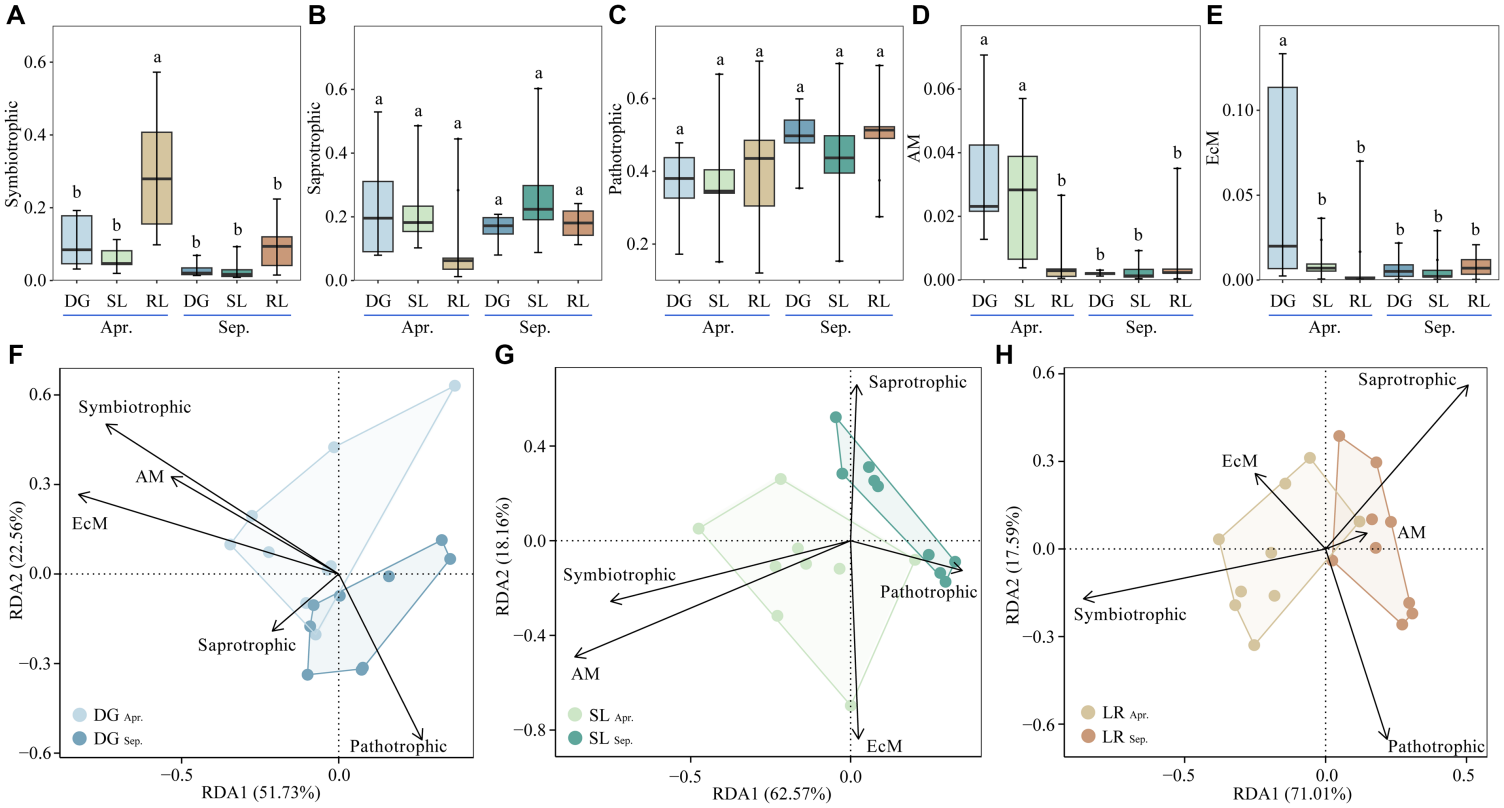
Relative abundance of soil fungal functional guild groups **(A–E)** and RDA analysis **(F–H)** under seasonal variation and land conversion in desert grassland. DG, desert grasslands plots; SL, shrub land plots; RL, land conversion plots. Apr, April; Sep, September.

Based on Funguild fungal function prediction, we classified fungal functional guilds into symbiotrophic, saprotrophic, pathotrophic, arbuscular mycorrhizal (AM) and ectomycorrhizal (EcM); and the differences in fungal functional guilds among the sample plot was analyzed using ANOVA. Finally, the interrelationships between fungal functional guild groups and environmental factors in the DG, SL and RL samples were determined by RDA at OTU level, following transformation by Hellinger method (Figure 6).

## 3 Results

### 3.1 Effects of land conversion on soil properties in desert grasslands

Conversion to agriculture profoundly affected soil pH, EC, total carbon (TC), total nitrogen (TN), total phosphorus (TP), total organic carbon (TOC), available nitrogen (AN), available phosphorus (AP), and available potassium (AK) contents; among these, soil electric conductivity (EC) increased significantly at the beginning of land conversion. With seasonal succession, soil pH and soil water content (SWC) also increased. Compared to desert grassland (DG) plots, both soil TN and AN content in shrubland (SL) and conversion (RL) plots significantly (*p* < 0.05) increased by seasonal succession (Supplementary Figure S1).

### 3.2 Effects of land conversion on soil fungal diversity in desert grasslands

Operational Taxonomic Units (OTUs), Shannon, and ACE indices of the fungal community were significantly reduced at the beginning of conversion to agriculture. In September, OTUs and ACE diversity indices in SL and RL plots were significantly lower than those in DG plots. In particular, seasonal succession significantly (*p* < 0.05) increased the number of OTUs and the ACE index values in DG plots, while reducing those in RL plots (Figures 2A–C). In addition, NMDS analysis revealed that the fungal communities in the experimental plots differed among groups in April and September (stress value <0.2) (Figure 2D).

Further, Mantel’s test showed that pH correlated negatively with EC, TC, TN, TP, TOC, AN, AP, and AK both in April and September. Further, in April, the alpha diversity index of the fungal community significantly correlated with pH, EC, TN, TP, AP, and AK. In contrast, in September, only the ACE diversity index significantly (p < 0.05) correlated with AK (Figures 2E,F).

### 3.3 Effects of land conversion on soil fungal community structure in desert grasslands

Ascomycota, Basidiomycota, Mortierellomycota, Glomeromycota, and Chytridiomycota were the dominant phyla of soil fungi in the study area (Figure 3A). The relative abundance of Glomeromycota significantly decreased during the early stages of land conversion. Further, seasonal succession had a significant effect on the relative abundances of Mortierellomycota and Glomeromycota in the soil. Particularly, the relative abundance of Mortierellomycota was higher in September than that in April, whereas Glomeromycota showed the opposite trend (Figure 3B).

Further analysis revealed that the dominant phyla of soil fungi showed different degrees of correlation with soil physicochemical properties in the early stages of land conversion (Supplementary Figure S2A), whereas, in September, only the relative abundance of Mortierellomycota was positively correlated with SWC (Supplementary Figure S2B). Moreover, redundancy analysis (RDA) revealed that SWC correlated significantly with Mortierellomycota, while EC significantly correlated with Ascomycota (Supplementary Figure S2C). Further analysis of the RDA of soil land patterns revealed that, in DG, the soil microbial in April was positively correlated with TC, TN, and TP. However, in SL, the soil microbial was negatively correlated with physicochemical properties while EC was positively with soil microbial in RL (Supplementary Figure S3A–C). In September, factors such as SWC, pH, AN, AK, and TOC were all positively correlated with the soil microbial community (Supplementary Figure S3).

### 3.4 Effects of land conversion on soil fungal-network relationships in desert grassland

Based on Spearman’s analysis, we constructed a co-occurrence network among fungal OTUs. The results showed that the nodes and edges of the fungal networks in RL plots were lower than those in DG and SL plots at the early stage of land conversion (Figures 4A–C). In contrast, in September, the nodes and edges of the networks in RL plots were higher than those in SL plots but lower than those in DG plots (Table 1, Figures 4D–F).

**Table 1:** Topological features of fungal networks under seasonal variation and land conversion in desert grassland.

Topological features	DG _Apr._	SL _Apr._	RL _Apr._	DG _Sep._	SL _Sep._	RL _Sep._
Total nodes	183	154	84	331	155	243
Total edges	205	138	68	313	148	231
Positive edges	130	84	38	179	76	153
Negative edges	75	54	30	134	72	78
Number of modules	40	44	23	63	42	58
Modularity	0.833	0.904	0.837	0.944	0.864	0.915
R square of power-law	0.972	0.952	0.906	0.856	0.941	0.91
Average degree	2.24	1.792	1.619	1.891	1.91	1.901
Average path distance	4.865	2.504	3.643	4.817	3.89	7.193
Geodesic efficiency	0.296	0.564	0.447	0.339	0.387	0.238

DG, desert grasslands plots; SL, shrub land plots; RL, land conversion plots. Apr, April; Sep, September.

The analysis of fungal network complexity revealed that SL plots had more complex fungal-network relationships in April, whereas in September, the fungal network complexity in RL plots was higher than that in SL or DG plots (Supplementary Figure S4). In the correlation analysis between NCI and environmental factors, at DG and SL plots in September, NCI had negative correlations with pH and SWC and positive correlations with TN, respectively. And in the RL plots, NCI was negatively correlated with TP (Figures 5A,B). Random forest analysis found that SWC had a high explanatory rate for NCI, followed by environmental factors such as AK, TN, and pH (Figure 5C).

### 3.5 Effects of land conversion on soil fungal functional guild groups in desert grassland

Among the five functional guilds, the relative abundance of symbiotrophic was higher at the RL plots than at the other plots in April (Figure 6A). The relative abundance of saprotrophic and pathotrophic were not significantly different among the plots (Figures 6B,C). In April, the relative abundance of AM in the DG plot was the highest, followed by that in the SL plot, and all of them were higher than other plots (Figure 6D). Similarly, the relative abundance of EcM was highest at the DG plots in April and was also higher than at the other plots (Figure 6E). Further analysis showed that symbiotrophic, AM and EcM had higher interpretation of the soil environment in the DG plots. In the SL plots, symbiotrophic and AM were more pronounced in indicating the soil environment. In the RL plots, symbiotrophic and Saprotrophic responded more positively to changes in the environment than the other functional guild groups (Figures 6F–H).

## 4 Discussion

### 4.1 Land conversion changed the soil fungal-community diversity in desert grassland

Conversion to agriculture profoundly affects soil physical properties, chemical structure, and soil microbial community (Hu et al., 2021; Kang et al., 2023). In this study, there were no significant differences in OTUs, Shannon, or ACE indices between DG and SL plots at the early stage of land conversion, whereas OTUs, Shannon, and ACE indices in RL plots were significantly lower than those in DG and SL plots. It has been shown that land conversion significantly reduces the diversity and richness indices of the soil fungal community, and it has also been suggested that desert grasslands are fragility and highly susceptible to human activities (Xu et al., 2017), largely because of the reduction in vegetation cover, which adversely affects the diversity of the soil fungal community (Cao et al., 2017). In the later stages of land conversion, there was no significant difference in the Shannon index of the soil fungal communities among the three treatment plots under study herein. More recent studies have suggested that, in general, land conversion has less of an effect on alpha diversity of fungal community than it does in the case of undisturbed desert soils (Hu et al., 2023). In addition, beta diversity analysis of fungal community further demonstrated the profound effects of different land-use practices on fungal community composition, consistent with previous studies (Li et al., 2017; Kang et al., 2023).

Interestingly, we found that the alpha diversity indices of fungal communities were significantly correlated with soil pH, EC, TN, TP, AP, and AK in April. However, seasonal variations (in September) reduced the significance of the relationship between soil fungal-community diversity and environmental changes. Soil fungal community diversity is highly influenced by environmental factors, such as pH and nutrients, and is subject to seasonal variation (Birgander et al., 2014; Hu et al., 2021). It can be speculated that, on one hand, soil fungal communities were more sensitive to environmental changes due to less precipitation in April, which likely strengthened their connections (Matulich et al., 2015), whereas, on the other hand, the increase in precipitation due to seasonal variations (including irrigation for conversion to agriculture), disrupted the dependence of fungal communities on environmental changes (Xu et al., 2017). In particular, the correlation between the ACE index and AK in September further indicated the importance of the effect of AK on the fungal community after land conversion (Slabbert et al., 2022). In summary, the land conversion from DG and SL to RL decreased the alpha diversity of fungi, with the fungal community structure experiencing alterations from a range of soil physicochemical properties in April, particularly being profoundly impacted by AK in September.

### 4.2 Seasonal succession was important in changing the soil fungal community in desert grassland

Soil fungal community composition in desert grasslands responds differently to land conversion and is primarily driven by changes in soil moisture and chemical properties (Hu et al., 2021). In particular, Ascomycota is an important group among soil fungi, and our RDA analysis revealed a strong correlation between Ascomycota, EC, and AP. Indeed, EC is the main factor influencing the relative abundance of Ascomycota (Yang et al., 2022). In turn, AP content in the case of disturbed grassland is also one of the major factors influencing the relative abundance of Ascomycota, whereby, it is not uncommon to find that the soil fungal-community structure under seasonal succession responds positively to changes in soil physical and chemical properties (Vargas-Gastélum et al., 2015). In addition, the relative abundance of the dominant phylum Mortierellomycota showed an upward trend influenced by seasonal succession. Consistently, a recent study pointed out that Mortierellomycota was significantly affected by seasonal changes, especially in SWC, which correlated positively with the relative abundance of Mortierellomycota (Yang et al., 2022). Hence, in this study, together with a gradual increase in SWC under seasonal succession, the relative abundance of Mortierellomycota might be used as an ecological indicator of soil moisture changes in desert grasslands.

Additionally, we found that the relative abundance of Glomeromycota decreased with seasonal succession. This might be explained by the fact that most members of the Glomeromycota (including AM) are profoundly affected by changes in the plant–soil system. In general, seasonal succession induces the input of plant litter into the soil, thereby increasing the number of Glomeromycota members (Ji et al., 2021; Kemmelmeier et al., 2022). We hypothesized that the increase in soil water content and available nutrients due to seasonal succession might have reduced plant dependence on soil nutrients, which in turn led to a decrease in the relative abundance of Glomeromycota (Zhang et al., 2023a,b). This result can be observed from the significant negative correlation between Glomeromycota members and TOC, AN, AP, and AK contents in April, indicating that soil fungal communities are regulated by seasonal changes.

### 4.3 The complexity of the soil fungal network was related to environmental and seasonal changes in desert grasslands

The complexity of soil fungal communities usually reflects the interrelationships among plants, environments, and the structure of the fungal community (Zhao et al., 2024). In this study, the number of edges and nodes in the RL plot network decreased during the early stage of land conversion, presumably because fungal communities are more susceptible to human disturbances, making changes in their network complexity more pronounced (Manning et al., 2023). We observed that the complexity of the RL plot network was significantly lower than that of SL plots; this finding agreed well with studies reporting that, when land is converted, the complexity of microbial networks is reduced (Cui et al., 2016; Chen et al., 2023). In September, the network complexity of RL plots was significantly higher than that of SL or DG plots. Soil nitrogen and phosphorus enrichment after land conversion significantly enhances the complexity of soil microbial community networks (Zhang et al., 2023a,b). In addition, the fungal network complexity in RL plots increased with seasonal succession, which supported previous findings suggesting that fungal network complexity increases with conversion time (Ma et al., 2021). These results indicated that in the initial stages of land conversion to agriculture, the soil fungal community was significantly disrupted. However, as cultivation continued and water and nutrients were introduced to the soil in later stages, the fungal community began to show signs of recovery and stabilization.

It is worth mentioning that seasonal changes have a strong effect on microbial network structure (Lan et al., 2022). However, in this study, the soil fungal network complexity in DG and SL plots did not significantly differ with seasonal succession. While on one hand, this finding indicates that soil fungal-network interrelationships in desert grasslands and shrublands are less affected by seasonal changes, on the other hand, it indicates that land conversion affected soil fungal-network interrelationships, because land conversion affects the physical and chemical properties of the soil, which in turn may affect soil microorganisms and, consequently, fungal network relationships (Lienhard et al., 2013). These results indicated the fragility of fungal communities in desert grasslands. Additionally, in our study, fungal network complexity in SL plots was consistently higher than that in DG plots, which further reflects the effect of desert grassland afforestation on soil fungal-network relationships. Complex network relationships generally indicate high stability of soil fungal communities, and contribute to the maintenance of soil ecosystem functions (Kang et al., 2023). Combined with previous studies, it is easy to find that additional water and nutrient inputs from agricultural land conversion have a positive effect on fungal network complexity. Meanwhile, the random forest analysis pointed out that SWC and AK were important environmental factors explaining the changes in NCI. Therefore, it further confirmed the previous studies and pointed out that the interrelationships among desert grassland fungi were influenced by SWC. The seasonal changes observed in the results validated that following land conversion to agriculture, the introduction of nutrients and water contributes to an increase in the network complexity of soil fungi. This means that additional resource investment may be one way to restore the ecosystem function of desert grasslands.

### 4.4 Functional groups of soil fungi in desert grassland responded positively to environmental changes

Land use change is one of the most important factors affecting the ecological function of soil fungi (Liu et al., 2023). The results showed that symbiotrophic was the functional guild indicative of environmental change in the three plots. It has been noted that symbiotrophic is more sensitive to changes in the land environment, including soil water content, carbon, and nitrogen nutrients are the main factors affecting changes in this group (Ren et al., 2023). Notably, symbiotrophic groups were significantly higher in the RL plots than in the other plots in April. This may be owing to the input of organic fertilizer (manure) during the conversion to agriculture process, which increased the relative abundance of symbiotrophic groups (Qiao et al., 2021). It has been suggested that the aggregation of saprotrophic groups is more sensitive to the transformation of land properties (Peng et al., 2019; Kang et al., 2023). The present study similarly found that saprotrophic groups were more closely related to soil physicochemical properties in the RL plots. In addition, the relative abundance of arbuscular mycorrhizal was higher in both DG and SL plots than in RL plots in April. On the one hand, we hypothesize that the DG plots served as the initial habitat for the colonization of AM groups and that habitat changes such as afforestation and land conversion have altered the original group aggregation (Rao et al., 2020). On the other hand, it has also been demonstrated that the response of AM functional groups to environmental changes such as soil SWC and pH can also serve as an important factor for predicting biodiversity in desert grasslands (Lüneberg et al., 2019; Wang et al., 2020). Different from previous studies, we found that the response of AM functional groups to environmental changes was not significant in the RL plots. A study showed that disruption of soil microbial communities by land conversion reduces AM response to environmental change (Holland et al., 2016). More studies have also concluded that the aggregation of AM functional groups is more sensitive to changes in plant diversity (Bahram et al., 2020). This study further confirms that changes in water and nutrient inputs into desert grasslands reportedly affect soil fungal communities and their functions in response to changes in soil ecological processes. The response exhibited by the functional groups of soil fungi during soil transformation suggests that resource investment may play vital roles in promoting the restoration of desert grasslands.

## 5 Conclusion

Conversion to agriculture has profound effects on the structure and function of fungal soil communities in desert grassland ecosystems. Land conversion significantly increased soil TC, TN, TP, AP, and AK contents. In the early stage of land conversion (in April), soil fungal OTUs and ACE indices were lower than those in DG and SL plots and significantly correlated with pH, EC, AP, and AK; moreover, the dominant phyla had strong correlations with soil physicochemical properties; concomitantly, the relative abundance of Glomeromycota was significantly lower, and the complexity of the fungal network in RL plots was lower than that in SL plots. Meanwhile, in the late stage of land conversion (in September), soil fungal OTUs and ACE indices were lower in RL plots than in DG plots, with more complex network relationships, compared to those in DG and SL plots. Symbiotrophic groups, a functional group of desert grassland soil fungi, can be used as a predictor of environmental change; in addition, land conversion decreases the relative abundance of AM functional groups. This study highlights the response of soil fungal communities and functions to human disturbances in desert grasslands. Considering the potential for land conversion to affect the secondary salinization of soils, there is a need for continued observation of soil ecological health under the time continuum of land conversion to agriculture.

## Data Availability

The datasets presented in this study can be found in online repositories. The names of the repository/repositories and accession number(s) can be found in the article/Supplementary material.
