# Supplementary material for: Response of soil fungal-community structure and function to land conversion to agriculture in desert grassland
Source: Front Microbiol. 2024 Sep 9;15:1413973. doi: 10.3389/fmicb.2024.1413973 (PMC11420991; doi:10.3389/fmicb.2024.1413973)
Supplement: Supplementary file 1 [file Data_Sheet_1.pdf]

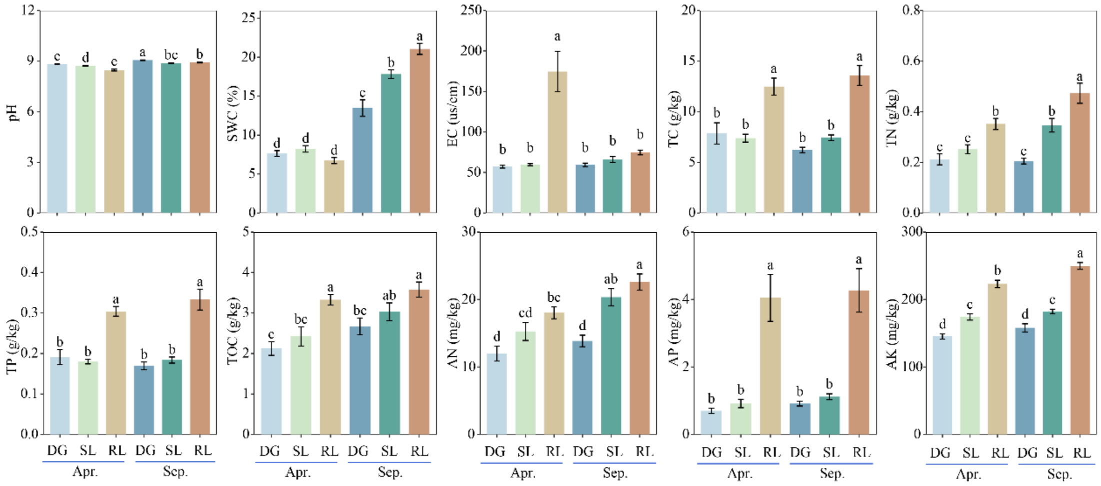

Figure S1. Soil physicochemical properties under seasonal variation and land reclamation in desert grassland. DG: desert grasslands plots; SL: shrub land plots; RL: reclaimed land plots. Apr: April; Sep: September.

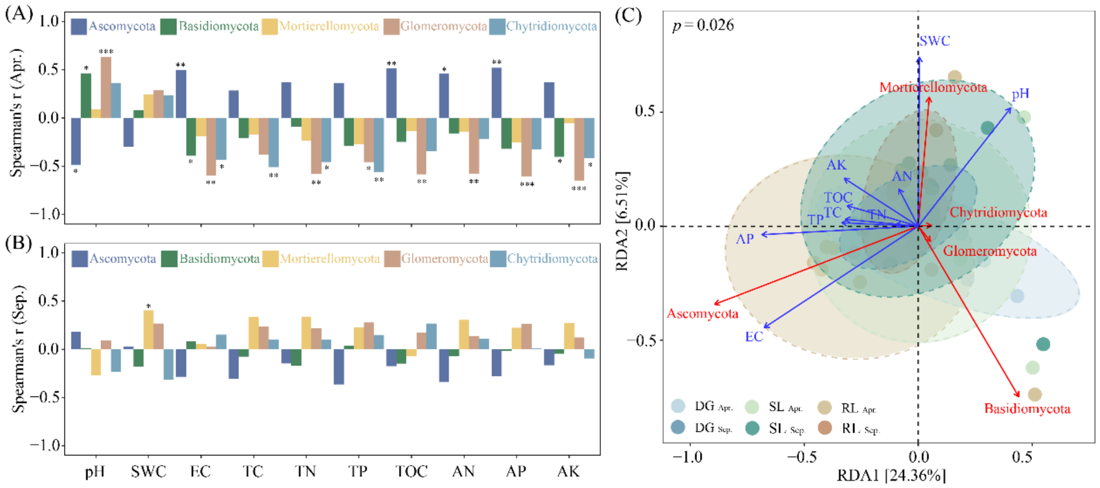

Figure S2. Correlation between dominant phylum of fungi and soil physicochemical properties (A: Apr; B: Sep), and RDA analysis (C) under seasonal variation and land reclamation in desert grassland. DG: desert grasslands plots; SL: shrub land plots; RL: reclaimed land plots. Apr: April; Sep: September.

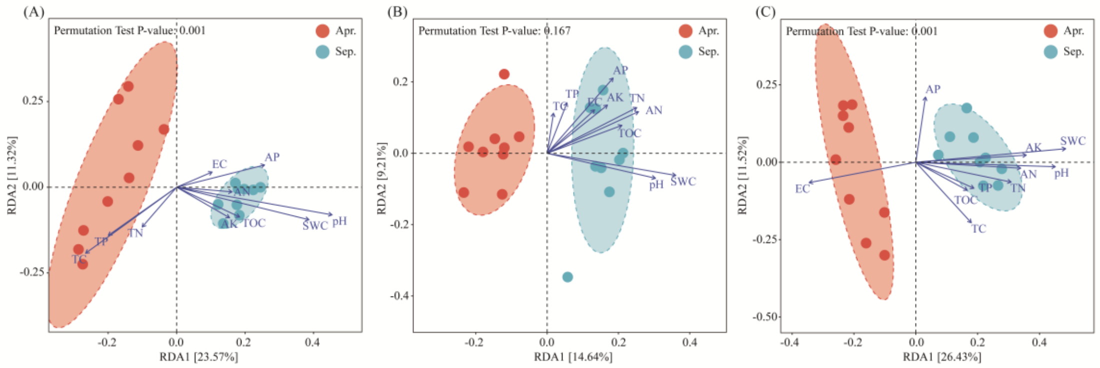

Figure S3. RDA analysis under (A) desert grasslands, (B) shrub land plots and (C) reclaimed land plots in April and September. Apr: April; Sep: September.

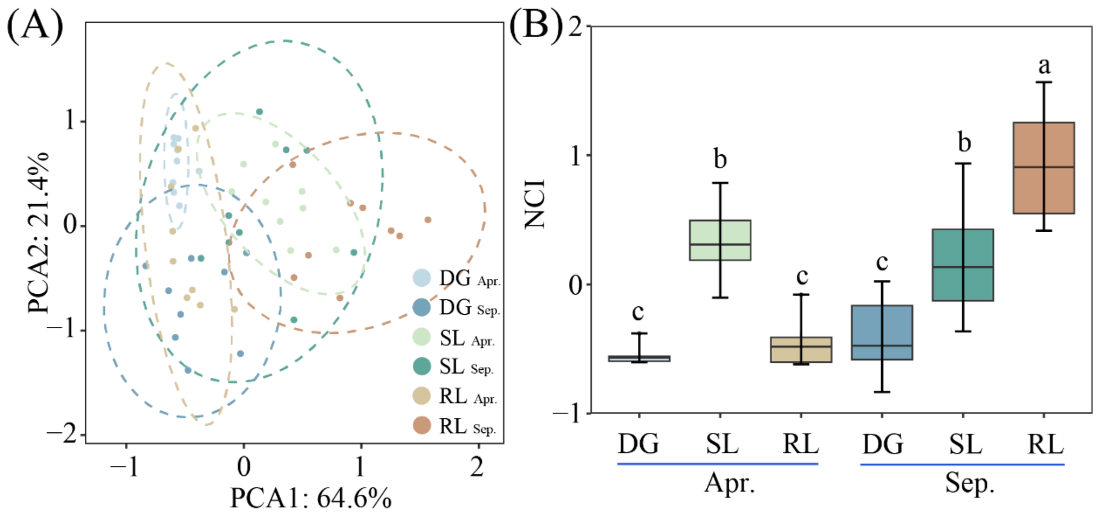

Figure S4. PCA analysis (A) and NCI index (B) of network parameters under seasonal variation and land reclamation in desert grassland. DG: desert grasslands plots; SL: shrub land plots; RL: reclaimed land plots. Apr: April; Sep: September.
